# Is the Capacity for Vocal Learning in Vertebrates Rooted in Fish Schooling Behavior?

**DOI:** 10.1007/s11692-018-9457-8

**Published:** 2018-06-13

**Authors:** Matz Larsson, Benjamin W. Abbott

**Affiliations:** 1 The Heart, Lung and Physiology Clinic, Örebro University Hospital, Örebro, Sweden; 2 School of Health and Medical Sciences, Örebro University, Örebro, Sweden; 3 Clinical Health Promotion Centre, Lund University, Lund, Sweden; 4 Department of Plant and Wildlife Sciences, Brigham Young University, Provo, USA

**Keywords:** Vocal learning, Incidental sound, Locomotion, Respiration, Locomotor-respiratory coupling, Fish schooling, Entrainment, Synchronization, FoxP2

## Abstract

The capacity to learn and reproduce vocal sounds has evolved in phylogenetically distant tetrapod lineages. Vocal learners in all these lineages express similar neural circuitry and genetic factors when perceiving, processing, and reproducing vocalization, suggesting that brain pathways for vocal learning evolved within strong constraints from a common ancestor, potentially fish. We hypothesize that the auditory-motor circuits and genes involved in entrainment have their origins in fish schooling behavior and respiratory-motor coupling. In this *acoustic advantages* hypothesis, aural costs and benefits played a key role in shaping a wide variety of traits, which could readily be exapted for entrainment and vocal learning, including social grouping, group movement, and respiratory-motor coupling. Specifically, incidental sounds of locomotion and respiration (ISLR) may have reinforced synchronization by communicating important spatial and temporal information between school-members and extending windows of silence to improve situational awareness. This process would be mutually reinforcing. Neurons in the telencephalon, which were initially involved in linking ISLR with forelimbs, could have switched functions to serve vocal machinery (e.g. mouth, beak, tongue, larynx, syrinx). While previous vocal learning hypotheses invoke transmission of neurons from visual tasks (gestures) to the auditory channel, this hypothesis involves the auditory channel from the onset. Acoustic benefits of locomotor-respiratory coordination in fish may have selected for genetic factors and brain circuitry capable of synchronizing respiratory and limb movements, predisposing tetrapod lines to synchronized movement, vocalization, and vocal learning. We discuss how the capacity to entrain is manifest in fish, amphibians, birds, and mammals, and propose predictions to test our acoustic advantages hypothesis.

## Introduction

Synchronized movement at organismal and group levels is ubiquitous in vertebrates, for example, schooling fish, swarming starlings, and ballet dancers. However, the ability to synchronize movement based on external auditory cues (entrainment) and the capacity to imitate sounds vocally (vocal learning) are much less common (Patel 2014). However, there is emerging evidence that entrainment and vocal learning are more widespread than previously believed (Condro and White 2014; Scharff and Petri 2011; Schachner et al. 2009; Wilson and Cook 2016; Arriaga et al. 2012). Entrainment appears to be linked with the capacity for vocal learning, although the mechanisms behind this relationship are hotly debated (Schachner et al. 2009; Patel et al. 2009; Patel 2014; Soma and Mori 2015; Merker et al. 2015).

At its most basic level, the ability to learn new vocalizations based on auditory input requires three related abilities: (1) perceiving and processing the relevant auditory signal, (2) temporally buffering the signal (i.e. committing it to memory), and (3) reproducing the signal with a complex motor system (in this case the vocal tract). Despite more than 300 million years of evolutionary separation, fish and tetrapods, including humans, share similar genetic factors (See: “FoxP2 in Vocalization”) and neuroanatomical structures for vocalization (Bass et al. 2008; Scharff and Petri 2011). Furthermore, all known vocal-learning vertebrates show behavioral, anatomical, and genetic convergence (Doupe and Kuhl 1999; Jarvis 2004; Nottebohm and Liu 2010; Pfenning et al. 2014; Condro and White 2014), with vocal learning closely associated with the neural and molecular mechanisms that regulate breathing, forelimb motor control, cerebral vocal control, and vocalization in the basal ganglia (Condro and White 2014; Feenders et al. 2008). Even the independently-evolved neurological hallmark of vocal learning—vocal control by telencephalic vocal nuclei (Feenders et al. 2008; Arriaga and Jarvis 2013)—shows striking similarities to control circuits in teleost fish (Forlano et al. 2014; Goodson and Bass 2002; Kittelberger and Bass 2013). Multiple gene factors and neural circuits shared by all vocal learners suggest strong genetic or epigenetic constraints on the evolution of this trait, potentially stemming from neuroanatomical structures inherited from a common ancestor (Jarvis 2004; Scharff and Petri 2011), either reptiles or fish.

The vocal learning and rhythmic synchronization hypothesis proposes that the ability to entrain to rhythmic auditory cues evolved as a by-product of vocal learning (Patel 2006; Schachner et al. 2009). It has also been proposed that entrainment evolved to attract mates, i.e. that vocal synchrony would amplify group broadcasting to distant females (Merker et al. 2009). Here we present an alternative hypothesis, that the ability to entrain movement to external sounds is prerequisite to the development of vocal learning and that some of the auditory-motor circuits and genes for entrainment and vocal learning have their origins in schooling behavior in fish. Because auditory perception plays a central role in fish synchronization (Pitcher et al. 1976; Faucher et al. 2010; Greenwood et al. 2013), molecular and neural mechanisms associated with schooling could have been co-opted for eventual entrainment and cerebral vocal control. In this paper, we develop this *acoustic advantages* hypothesis, specifically exploring how incidental sounds of locomotion and respiration (ISLR) could provide selective pressure to synchronize respiration and movement at the individual and group levels. We discuss how the capacity to entrain is manifest in fish, amphibians, birds, and mammals, and propose several experiments to test the acoustic advantages hypothesis. While no species basal to both teleost and tetrapod lineages still exist, we compare genetic, neural, and behavioral characteristics of vertebrates across these groups to assess likelihood of deep homology.

### A Spectrum of Entrainment and Vocal Learning

Entrainment and vocal learning have typically been defined as categorical abilities expressed by a narrow group of cognitively advanced animals (e.g. Merker et al. 2009, 2015; Patel et al. 2009). While qualitative differences may exist between vocal learners and non-learners (Jarvis 2004), much of the binary view of entrainment may be an experimental artefact (Scharff and Petri 2011; Arriaga et al. 2012). The few animals that have been tested for entrainment have typically been classified categorically based on whether they synchronize movement to human music or rhythmic sound (Arriaga et al. 2012; Petkov and Jarvis 2012). While entrainment to artificial sounds allows a relatively objective classification, interpreting the evolutionarily relevance of this metric is not straightforward (Petkov and Jarvis 2012). Music and dance are influenced by culture, indeed they are products of culture, and major structural features of music and responsiveness to music are shaped by the cultural transmission process itself (Merker et al. 2015). For example, depending on the choice of music, the cultural background of the subject, and the degree of musical experience, it is likely that some humans would not entrain when exposed to some types of music. This cultural mismatch becomes orders of magnitude more problematic when applied across species (Bolhuis and Wynne 2009; Petkov and Jarvis 2012). Exposure to rhythmic sound that an animal would naturally experience (e.g. flapping of wings, swimming sounds, or footfalls) could offer a more appropriate test of entrainment abilities, though entrainment and vocal learning are admittedly difficult to quantify across diverse lineages.

In contrast to the categorical view, there is a growing perspective that motor and vocal synchronization and imitation exist on a continuum (Arriaga et al. 2012), ranging from phase matching (e.g. coordination of firefly flashes) to complex learned behaviors involving listening, practice, and performance (e.g. bird, whale, and human song; Wilson and Cook 2016; Scharff and Petri 2011; Petkov and Jarvis 2012). In this view, some level of entrainment and adaptability of vocalization is the norm among vertebrates and even some invertebrates. At one end of the spectrum, simple forms of temporal synchrony arise spontaneously due to sensorimotor coupling. At the other end, humans achieve predictive timing that features both phase and period correction, so-called “entrainment with perfect synchrony” or “negative asynchrony” where the response precedes the call, depending on advanced telencephalic learning (Merker et al. 2015, 2009). Notably, only a few species have indisputably been shown to be unable to exhibit vocal learning (Kroodsma and Konishi 1991), and unrecognized intermediary phenotypes between accurate imitative ‘production’ learning and ‘usage’ learning may exist (Janik and Slater 2000). We use this continuum hypothesis (Arriaga et al. 2012; Arriaga and Jarvis 2013) to explore temporal coordination associated with neuroanatomical and behavioral characteristics relevant to vocal learning as expressed in modern tetrapod lineages, including respiratory-motor coupling, forelimb motor processing, and sound-producing movements occurring during group locomotion.

### Which Came First, Entrainment or Vocal Learning?

**Fig. 1:**
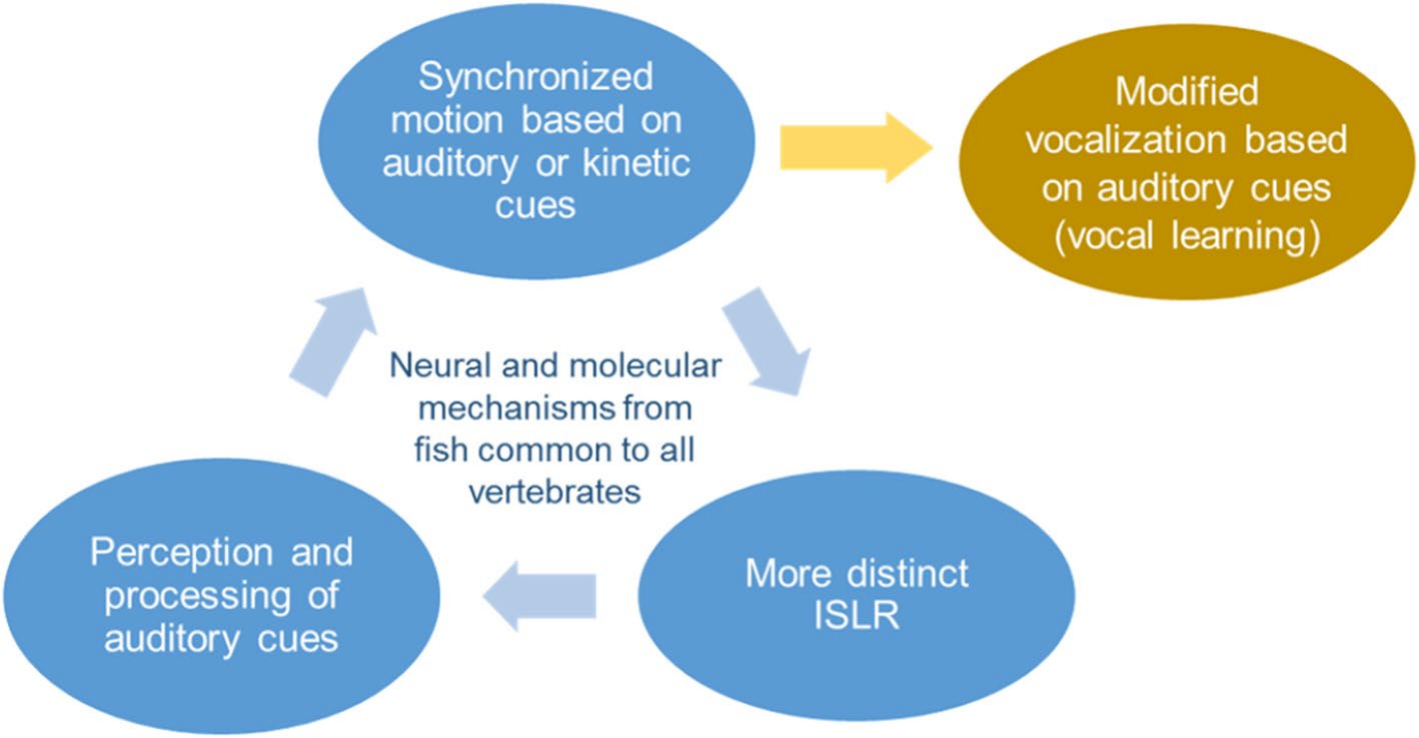
Conceptual model of the acoustic advantages hypothesis. The ability to synchronize movement based on external auditory cues (entrainment) could have evolved partially to minimize auditory masking in fish schools and confuse predator’s octavolateralis system. This synchronization results in organized ISLR, which improves situational awareness of the individual and the group. The neural circuitry and genetic factors linking sound, movement, and respiration, which evolved in fish, could then have been co-opted for vocal synchronization in mammal and avian species capable of vocal learning

While it has been hypothesized that the ability to learn vocally is prerequisite to the ability to entrain to external acoustic cues, i.e. that vocal learning came first (Patel et al. 2009), entrainment has been observed in a large number of species that lack vocal learning abilities, including sea lions, frogs, and fireflies (Cook et al. 2013; Condro and White 2014; Wilson and Cook 2016; Ravignani et al. 2014). Additionally, auditory turn taking and call and response behaviors (arguably direct precursors to entrainment) are widespread in tetrapod lineages (Bee 2007; Borjon and Ghazanfar 2014; Louwerse et al. 2012). Before entrainment or vocal learning can occur, there must be awareness and responsiveness to the sonic environment (Fig. 1).

The ability to perceive and interpret complex sonic signals has a host of benefits, including facilitating social interactions (Wallin et al. 1999; Fitch 2010; Nagasaka et al. 2013; Bee 2007), and numerous individual and group acoustic advantages such as predator evasion and increased situational awareness (Larsson 2009, 2012b, 2012a, 2013, 2014; Vanesyan et al. 2015). To make the evolutionary leap from sonic awareness and motor entrainment to vocal learning (defined in the limited sense of being able to learn and reproduce vocalizations), auditory-motor circuits would need to be co-opted for vocal control (Fig. 1). Molecular and neural mechanisms originally linking respiration with locomotion could have been readily exapted for this purpose because many structures in and surrounding the vocal tract and lungs are homologous to respiratory anatomy in fish, such as gill arches and the swim bladder (Fitch 2002; Bass and McKibben 2003). Indeed, both hearing (Ladich and Popper 2004) and the use of respiratory structures for vocalization extend back to fish, with some species using the swim bladder and other respiratory structures for sound production (Connaughton et al. 2002; Bass et al. 2008), potentially to locate and assemble with conspecifics (Kuznetsov 2009). In addition to the anatomical homology in vocal tracts, fish and tetrapods share deep molecular similarities. Genetic factors essential to vocalization and vocal learning in tetrapod lineages, such as the Forkhead box proteins FoxP1 and FoxP2 (see “Fact Sheet”; Lai et al. 2001; Wohlgemuth et al. 2014), are also present in fish, where they regulate analogous social and learning behaviors and development of the central nervous system (Condro and White 2014; Roberts et al. 2013; Scharff and Petri 2011; Bonkowsky et al. 2008).

Given the evidence of a possible ichthyic origin of entrainment and other precursors to vocal learning in tetrapods (Bass et al. 2008; Condro and White 2014; Roberts et al. 2013), we use the following sections to explore the selective pressures that could have led to the evolution of schooling and motor-respiratory coupling in fish. First, we explore genetic data relevant to the hypothesis, focusing on the transcription factor *FoxP2*, which has been investigated in a diverse range of species. Second, we consider the neuromolecular basis and selective advantages of fish synchronization in general. Third, we examine the specific case of locomotor-respiratory coupling, which is particularly relevant to vocalization in air-breathing vertebrates (Bardy et al. 2015; Fabre et al. 2007). We adopt an aural approach in these investigations, in an attempt to understand how aquatic organisms such as fish experience their cacophonous environment. We use this auditory perspective to hypothesize origins of the genetic factors, neural circuits, anatomical structures, and social behaviors that underpin vocalization and vocal learning in modern tetrapod lineages.

### FoxP2 in Vocalization

A gene on chromosome 7 called Forkhead box P2, or FOXP2 (Lai et al. 2001) (see “Fact Sheet”) is essential to human language development, associated with structure of the striatum, cerebellum, and cortex (Lai et al. 2001; Vargha-Khadem et al. 2005; Mori and Wada 2015). Humans that are heterozygous for a non-functional FOXP2 allele experience impairment in speech and language development (Lai et al. 2001; Graham and Fisher 2015). One symptom is that they perceive rhythmic differences in stimuli less well and imitate rhythms less accurately than control subjects (Alcock et al. 2000). FOXP2 is proposed to play a crucial role for learned vocal-motor behavior such as singing and speech (Ayub et al. 2013).

FOXP2 affects dopamine-dependent learning processes in specific regions of the striatum (Schreiweis et al. 2014; Raghanti et al. 2016), though mice carrying only one functional *Foxp2*, show additional and partly opposite effects (Enard 2011), suggesting that FOXP2 has contributed to tuning cortico-basal ganglia circuits during the evolution of human speech and language (Enard et al. 2009; Schreiweis et al. 2014). Notably, the two amino-acid coding changes that differentiate the human sequence from that of chimpanzees were also present in Neandertal and Denisovan individuals (Krause et al. 2007; Enard 2016).

### Fact-Sheet

The name “Forkheadbox” was coined in 1989 when fruit fly researchers identified mutants with a «forked head» and they connected this phenotype to a specific stretch of DNA, called box (Weigel et al. 1989). This “forkhead box”, subsequently shortened to “Fox”, consists of more than forty genes organized into nineteen gene families: *FoxA, FoxB, …FoxS*.* These genes code for proteins regulating the function of other genes, in other words they are transcription factors that reduce or enhance activity by binding to specific regulatory regions of target genes. Several Fox transcription factors affect health and disease, but only the *P* gene family has been linked to speech and language (Wohlgemuth et al. 2014). Typically, genes have multiple roles at numerous developmental time points or in different environmental contexts, for example, the ~ 20,000 protein-coding genes in the human genome are “re-used” in a number of different contexts in the brain and body (Fisher 2017). The pathways by which genetic factors influence neural circuitry and cognitive functions are indirect and require intermediate mechanisms such as proliferation, differentiation, connectivity, and plasticity. A gene cannot specify a precise behavior output, or a particular neural circuit, thus to discuss a “gene for language” is not particularly constructive (Fisher 2017). Likewise, *FoxP2* fills multiple roles. It influences the development of neural circuitry engaged in sensory guided motor learning, such as dendritic outgrowth and spine formation, and also the proper function of these circuits (Wohlgemuth et al. 2014). *FoxP2* is also important in lung development, thus the *FoxP2* gene is neither unique to humans nor exclusively associated with the central nervous system (Schatton and Scharff 2017).

*When capitalized, *FOXP2* denotes the human gene, *Foxp2* = the gene in mice, and *FoxP2* = all other species (Kaestner et al. 2000). The genes are italicized (END OF FACT SHEET).

*FoxP2* exist in all 274 vertebrates studied so far (Scharff and Petri 2011), and is among the best conserved genes throughout vertebrate evolution, suggesting strong evolutionary pressure (Schatton and Scharff 2017). *FoxP2* has ancient roles in the growth and function of brain circuits in the cortex, basal ganglia and cerebellum, with bearing on sensorimotor integration and motor-skill learning (French et al. 2012; Scharff and Petri 2011; Fisher 2017). During embryonic development, *FoxP2* is expressed in homologous brain regions, including the basal ganglia, of human, monkeys, various rodent species, different bird species, frogs, and fish (Scharff and Petri 2011). In songbirds and mammals, the *FoxP2* protein is expressed in medium spiny neurons of the striatum, a region important for translating sensory stimuli (auditory in humans and birds, visual and tactile in mice) into motor acts (speech in humans, song in birds, and locomotion in mice; Schatton and Scharff 2017).

Beyond its developmental functions, *FoxP2* regulates the song circuitry in vocal-learning songbirds, hummingbirds, and parrots (Feenders et al. 2008). These three orders are not linked by an immediate common ancestor (Hackett et al. 2008) and it is unclear whether they evolved the neural circuitry for vocal learning independently, or if a common ancestor to most extant birds possessed this trait that was more or less lost in non-vocal learners (Scharff and Petri 2011). *FoxP2* and its related molecular network have been proposed as evolutionary constraints contributing to convergent evolution of learned vocal communication in diverse taxa (Scharff and Petri 2011).

### Dopamine

In addition to its direct effects on brain function, *FoxP2* may be indirectly linked with vocal learning via interactions with the neurotransmitter dopamine. Experimental evidence in mice and birds suggests that *FoxP2* and its associated molecular network interact with dopamine signaling to regulate the strength of connections between particular sets of neurons, effectively fine tuning sensory-motor integration (Wohlgemuth et al. 2014). The basal ganglia have similar form and function in avian and mammalian brains, suggesting that sensory-motor learning in this circuit may rely on neurochemical rewards including dopamine transmission (Schatton and Scharff 2017). Auditory stimuli such as music listening stimulates dopamine release in the dorsal and ventral striatum in humans (Meehan et al. 2018; Zatorre 2015) and other animals (Panksepp and Bernatzky 2002; Sutoo and Akiyama 2004; Mavridis 2015). Dopamine may additionally aide in discrimination of important signals (Durstewitz et al. 1999), including footsteps and other ISLR, conveying adaptive value and contributing to the evolution of rhythmic abilities (Larsson 2014).

### Acoustic Benefits of Synchronized Movement for Fish

The occurrence of synchronized movements in all classes of vertebrates (Krause and Ruxton 2002; Larsson 2012a, 2013, 2014) suggests that the vertebrate brain has an innate capacity for kinetic coordination in groups, though this attribute may only be expressed under the right behavioral and ecological conditions (Larsson 2012a; Greenwood et al. 2013). Synchronized group movement in fish and other animals may have developed to improve foraging, hydro or aerodynamics, decision making, or predator evasion (Foster et al. 2001; Krause and Ruxton 2002; Svendsen et al. 2003; Parrish and Edelstein-Keshet 1999; Berdahl et al. 2013). Fish experience an intensely aural world (Buerkle 1969; Slabbekoorn et al. 2010), sensing pressure waves with an advanced octavolateralis system consisting of the ear and lateral line, a pressure-sensitive organ running the length of their body (Coombs and Van Netten 2005). The octavolateralis system also provides mechanosensory input (i.e. sensing of water motion and electromagnetic cues for some fish), which is more or less analogous to hearing in different fish clades (Butler and Maruska 2016a, b). Consequently, acoustic advantages of schooling could have complimented or preceded other selective pressures, contributing to the emergence of sonic awareness and entrainment.

The majority of fish species school at some point in their development (Shaw 1978), though the type and synchrony of schooling depend on socioenvironmental conditions and species traits, including neurological and genetic factors (Greenwood et al. 2013; Suriyampola et al. 2016). The ability to school requires two of the three prerequisites for vocal learning, i.e. perceiving and filtering auditory information and coupling that stimulus to complex movement (Fig. 1). While visual cues can strengthen schooling behavior (Rowland 1999), the molecular and neurological basis for schooling appears to be aural, since blind or blinded fish still school (Pitcher et al. 1976; Faucher et al. 2010; Kowalko et al. 2013) but fish with a temporarily disabled lateral line only do with great difficulty (Partridge and Pitcher 1980; Faucher et al. 2010).

One of the fundamental advantages of schooling is protection from predators (Shaw 1978; Greenwood et al. 2013). The protective benefits of schooling have primarily been attributed to visual confusion (Pavlov and Kasumyan 2000). However, many predatory fish rely heavily on their octavolateralis system (Moulton 1960; Larsson 2009) for prey detection (Montgomery and Bodznick 1999), particularly in the final stages of an attack (New et al. 2001). Consequently, to avoid being eaten, fish must thwart both the visual perception of a predator (Ruxton et al. 2007; Berdahl et al. 2013), and its auditory awareness (Larsson 2009). The latter could have been the primary or initial protective advantage of schooling in fish, i.e. to create complex, overlapping sound and pressure waves that confuse the predator’s octavolateralis system (Larsson 2009, 2012b).

While sound from a school may confuse a predator, sounds from a solitary fish can attract predatory attack (New et al. 2001). One possible selective advantage of strongly synchronized ISLR, would be to reduce a predator’s ability to identify the number of individuals based on sound alone. For example, in species that synchronize movement and respiratory sounds, a group may sound like an individual, and an individual like the group, potentially creating hesitation in predators and conveying secondary protection. Together, the benefits of being able to confuse a predator’s inner ear, lateral line, and electrosensory system may have strengthened selection for individuals with the ability to synchronize their movements and respiration to external sound (Larsson 2009, 2012b).

Another advantage of synchronized movement is improved auditory perception of the school due to coordinated onset and cessation of ISLR (Larsson 2009). Noise from a fish’s own movement or from external biotic and abiotic sources can drown out pertinent environmental sounds such as noise from predators or prey (Buerkle 1969; Slabbekoorn et al. 2010). Temporally coordinated movement and respiration in a school could reduce auditory masking because synchronous onset of noise improves auditory grouping (Bregman 1990) and synchronous cessation of movement creates momentary windows of silence, increasing situational awareness by allowing the group to hear predators, prey, and signals from nearby conspecifics (Larsson 2009).

Within a school of fish, ISLR may also play a communicative function, broadcasting information about location and speed (Moulton 1960; Pitcher et al. 1976), and even fitness and social status (Butler and Maruska 2016b). ISLR produced by nearby school members may act as external auditory cues for group synchronization, linking auditory stimuli with motor control (Larsson 2012a, 2014). Since these are the building blocks for the capacity to entrain, the neural and genetic framework for schooling, which is present in cartilaginous and bony fish, could later increase likelihood of the evolution of synchronized vocalization and even vocal learning in some tetrapod species (Bee 2007; Roberts et al. 2013; Wohlgemuth et al. 2014). The communicative function of ISLR could result in an evolutionary positive feedback, with better synchronization resulting in more precise ISLR and in turn improving synchronization (Fig. 1). This feedback would rapidly increase the signal to noise ratio of ISLR, conveying selective advantage to individuals with neural circuitry and genes capable of deciphering complex auditory signals and linking this information with motor control (Lieberman 2001). The fact that reaction times to auditory stimuli are 14–40% faster than for visual stimuli in mammals and birds (Whitchurch and Takahashi 2006; Pain and Hibbs 2007; Shelton and Kumar 2010) could be a consequence of the highly developed auditory-motor circuits inherited from fish.

### Schooling Formations May Favor Acoustic Awareness

It has long been believed that that fish school to swim more efficiently (Weihs 1973). Faster swimming fish do group closer together and swim with greater synchrony (Ashraf et al. 2017). In a study of fish pairs, 90% of the pairs were synchronized when swimming at high speed (Ashraf et al. 2016). This tendency of fish to swim closer and synchronize movements more at high speed has been attributed solely to energy gain, though acoustic advantages could provide a complimentary advantage. Indeed, the energy gain hypothesis has been based more on theoretical modelling than observations from nature (Ashraf et al. 2017), and there are several lines of evidence that school architecture is not optimized for swimming efficiency. In several species, schooling fish do not swim in appropriate positions to maximize hydrodynamic advantage (Partridge and Pitcher 1979). Schools of the cohesive fish species *Hemigrammus bleheri* tend to gather in a phalanx configuration (side by side, similar to a military formation) when swimming fast, though an echelon formation would be energetically more efficient (Ashraf et al. 2017). We suggest that an alternative reason for swimming in phalanx could be improved perception of neighbors’ ISLR. Individual’s ears and lateral lines will be relatively close and water-movements and sound will be relatively symmetrically propagated when swimming side by side. Moving close to one another, ISLR will be transmitted faster between fish and ISLR will be of larger amplitude, two things that are likely to reinforce synchronization based on acoustic cues. This hypothesis is supported by observations that phalanx formation is associated with local kinematic synchronization of each swimmer with its nearest neighbors (Ashraf et al. 2017).

### Evolution and Perceptual Consequences of Locomotor-Respiratory Coupling

While many of the physiological, molecular, and neural functions used during entrainment and vocal learning in tetrapods are present in fish, several notable changes in auditory-motor coupling have occurred since these groups diverged some 370 million years ago. Specifically, the lateral line, which is ineffectual in air, was lost in terrestrial tetrapods, and mouth breathing resulted in increased coupling of sound production and respiration (Bass and Chagnaud 2012). While locomotor-respiratory coupling was already present in fish (Tytell and Alexander 2007; Hoffman et al. 2016), air breathing necessitated deeper integration of central vocal motor commands into the respiratory cycle as well as somatosensory feedbacks in respiratory and vocal motor pathways (Smotherman et al. 2006). For example, in modern birds, the vocal motor system may occasionally take control over the respiratory musculature through central pathways, but pulmonary and laryngeal proprioceptive feedbacks usually allow interweaving vocalizations with breathing (Suthers et al. 2002). The parabrachial nucleus is a central constituent of the mammalian vocal motor pathway (Smotherman et al. 2006), with the lateral region strongly influencing respiratory rhythms and locomotor-respiratory coupling in mammals and other non-mammalian tetrapods (Bramble and Carrier 1983). Coordination of laryngeal and respiratory activity is synchronized through a network of interacting medullary and pontine feedback pathways—the same network that regulates breathing, chewing, swallowing, drinking, vomiting, coughing, and sneezing (Sakamoto et al. 1997). These fundamental functions long predate the exaptation of the system for vocal communication in terrestrial tetrapods (Smotherman 2007), in some cases extending back to fish ancestors (Perry et al. 2001). Together, the neural circuits regulating locomotor-respiratory pathways in fish provide a plausible neurological basis for the association between forelimb motor processing and vocalization in tetrapods (Bass and Chagnaud 2012).

#### Locomotor-Respiratory Coupling in Individual Fish

**Fig. 2:**
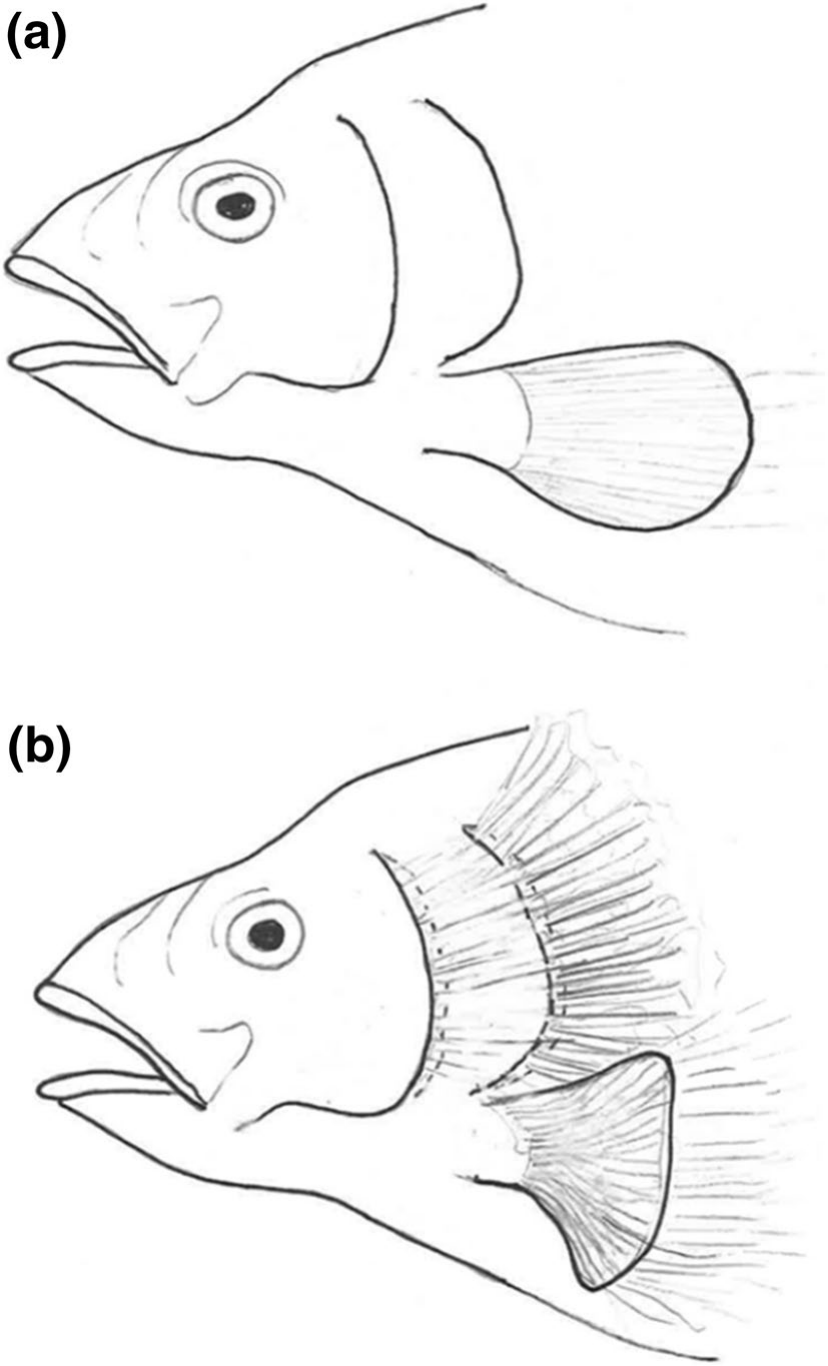
Drawing of locomotor-respiratory synchronization in bluegill (*Lepomis macrochirus*) during pectoral fin abduction (**a**) and during pectoral fin movement and operculum pumping (**b**). Lines in (**b**) represent turbulence and sound associated with respiration and fin movement. Bluegill and other fish synchronize locomotion and ventilation, potentially for auditory reasons. The synchronization of these two movements minimizes interaction between the flow from the operculum and flow over the pectoral fins, creating windows of silence and improving situational awareness

The synchronization of respiratory rhythms with locomotor patterns is evident in all classes of vertebrates (Bramble and Carrier 1983; Funk et al. 1992) and has been hypothesized to reduce energy consumption by avoiding competing muscle groups from working against one another (Bramble and Carrier 1983). However, energy gain by locomotor-respiratory coupling seems to be insignificant in humans (Wilke et al. 1975) and fish (Tytell and Alexander 2007), though there is some evidence to the contrary (Akanyeti et al. 2016). A non-mutually exclusive hypothesis is that acoustic benefits contributed to the evolution of locomotor-respiratory coupling. *Lepomis macrochirus* (Bluegill) tend to ventilate the gills every second or third pectoral fin beat, with a regular phase relationship between locomotion and ventilation (Tytell and Alexander 2007). During pectoral fin abduction, the pumping operculum (the hard bony flap covering the gills) creates a jet of water that stops immediately after the fin is fully abducted (Fig. 2).

Abduction of the pectoral fin is likely to produce more intense masking sound, particularly during fast swimming, while in contrast, adduction of the fin will result in a more streamline fish body and consequently reduced turbulence. The synchronization of these two movements reduces interaction between flow from the operculum and flow over the pectoral fin, minimizing turbulence (Tytell and Alexander 2007) and likely reducing noise (Larsson 2012a). Notably, the pectoral fin operates adjacent to the inner ear. Altogether, locomotor-respiratory coupling in bluegill results in concurrent respiratory and movement turbulence (Tytell and Alexander 2007) and consequent noise, which prolongs quite intervals, reducing masking problems for an individual fish. There is recent evidence that salmonids (and likely other species) coordinate respiration and motion to improve auditory perception (Akanyeti et al. 2016), supporting the hypothesis that acoustic advantages played a role in the evolution of locomotor-respiratory coupling. Additionally, mechanosensory neurons in teleost fish and second-order electrosensory neurons in elasmobranch fish can regulate to cancel the effects of stimuli that are tied with fish respiratory movements (Montgomery and Bodznick 1994).

#### Locomotor-Respiratory Coupling in the School

Little is known about respiratory behavior in schools, and it is unknown if fish synchronize respiration to elongate relatively quiet intervals. Operculum jet turbulence and other ISLR from individual fish are likely to be perceived by nearby conspecifics, potentially masking other environmental sounds. At the same time, the jet may communicate important information, about distance, speed, and position of nearby fishes, which may help fish maintain accurate distance and avoid collisions (Butler and Maruska 2016b). Since the operculum jet is phase locked with the sound of pectoral fins, the operculum jet may serve as an auditory cue reinforcing synchronization of fin movements, and vice versa. This locomotor-respiratory neural circuitry could potentially have been co-opted for auditory entrainment from external cues in tetrapod descendants. Notably, vocal and pectoral systems seem to have a shared developmental origin. So far all investigated tetrapods have forelimb motor neurons that serve in both sonic and gestural signalling (e.g. the pectoral fin of midshipman fish) (Bass and Chagnaud 2012). For some species this could be directly connect with vocalization such as for many Gobiidae species that use a pectoral-girdle-based mechanism for sound production (Parmentier et al. 2017). Similarly, herring and whiting emit sound signals, ostensibly from the swim bladder, before rapid maneuvers (Gray and Denton 1991).

#### Locomotor-Respiratory Coupling in Other Vertebrates

There is some evidence from other tetrapods that auditory advantages are associated with ISLR and synchronization of respiration and locomotion. Flocks of geese vocalize intensely before departure; their movements and vocal signals seem to act in combination as communicative cues (Ramseyer et al. 2009). Goose vocalization is amplified breathing sound and since breathing is phase-locked with wing flapping (Funk et al. 1993), these vocal calls inherently carry rhythmic information about location and trajectory, potentially reinforcing synchronization and the link between entrainment and external auditory cues. Vocalization also crescendos during take-off and once in flight before climbing, supporting the idea that this respiratory noise plays a communicative role. Many bird species, such as sparrows (*Passer domesticus*) and jackdaws (*Corvus monedula*), vocalize continuously when flying in formation. Breathing in dolphins is necessarily explosive (Fahlman et al. 2015), producing considerable turbulence and noise, and both diving dolphins (Hastie et al. 2003) and whales (Senigaglia and Whitehead 2012; Aoki et al. 2013) display synchronized breathing, potentially to reduce problems of auditory masking. Humans tend to synchronize breathing in groups unconsciously, a tendency that is reinforced by external rhythmic stimulation such as music listening (Codrons et al. 2014) or hearing a metronome (Bardy et al. 2015).

## Discussion

We hypothesized that the neural circuitry, genetic factors, and anatomy originally used in fish synchronization provided constraints on the evolution of entrainment and vocal learning in tetrapods, and specifically that locomotor-respiratory coupling in fish is linked with forelimb motor processing and vocalization in tetrapod descendants. Acoustic benefits of locomotor-respiratory coordination in fish may have selected for genetic factors and brain circuitry capable of synchronizing respiratory and limb movements, predisposing tetrapod lines to synchronized movement, vocalization, and vocal learning. Neuromolecular commonalities among tetrapods and fish and the strong link between forelimb motor processing and vocal communication in all investigated vertebrates support these hypotheses (Bass and Chagnaud 2012; Feenders et al. 2008; Scharff and Petri 2011).

Acoustic advantages may have also been one of the original benefits of social interaction for fish (Larsson 2012b), another factor increasing the likelihood of the evolution of auditory entrainment. Synchronization of movements during schooling may: (1) reduce masking problems caused by ISLR, (2) improve the signal function of ISLR within the school, and (3) confuse the lateral line and electrosensory perception of predators (Larsson 2009, 2012b, 2013). Our analysis of the literature indicated that synchronization of breathing and locomotion in individuals (i.e. locomotor-respiratory coupling) may also result in improved auditory perception—an alternative mechanism to the energetic efficiency hypothesis (Wilke et al. 1975; Tytell and Alexander 2007).

### Why are There So Few Vocal Learners?

If the roots of vocal learning extend all the way back to fish, another question emerges: why do so few vertebrates express vocal learning (Fitch 2011)? All tetrapods are descended from fish, however only a small fraction appear to have the capacity for vocal learning or entrainment based on external rhythmic cues (Patel 2014; Merker et al. 2015). Darwin’s hypothesis that movement in response to rhythmic auditory stimuli was likely universal (Darwin 1871/1981) has not been borne out by the data, though only a fraction of all species have been tested for these abilities (Scharff and Petri 2011). A telencephalic explanation of the infrequency of vocal learning does little to resolve the vocal learning conundrum, because many highly encephalized vertebrates lack complex vocal learning abilities. Given the potential benefits of vocal learning and the diversity of taxa that show some version of call imitation, it is puzzling that some degree of vocal learning has not evolved in more species (Sewall et al. 2016), particularly in cognitively advanced, social mammals such as dogs, hyenas, lions, and non-human primates.

One null solution to the vocal learning conundrum is that entrainment and vocal learning are simply much wider spread than currently believed (Condro and White 2014; Patel 2014). Testing more species with more appropriate methods may reveal a spectrum of abilities better distributed across lineages (Scharff and Petri 2011) in line with the continuum hypothesis (Arriaga and Jarvis 2013). However, if entrainment and vocal learning prove to be rare after more exhaustive study, multiple selective pressures could account for this sparse distribution. One possibility, is that the specialized neural mechanisms that underpin vocal learning (Bolhuis and Wynne 2009; Chakraborty and Jarvis 2015; Jarvis 2004) may simple be difficult to evolve on a structural or technical level (Chakraborty and Jarvis 2015; Isler and van Schaik 2006; Mink et al. 1981). Another possibility is that tradeoffs associated with vocal learning could have prevented the evolution of this trait in certain groups. The functional costs of learning processes, including time and social retaliation for making errors (Akcay et al. 2012; Sewall et al. 2016), can be avoided if unlearned vocalizations are sufficient for mediating social dynamics, such as when species live in social groups that are small, stable, and genetically homogenous. In such situations, unlearned calls, or calls learned during a single critical period, can function to mediate social interactions within and among groups without incurring costs associated with vocal learning (Seyfarth and Cheney 2014). It has been proposed that vocal learners could be more vulnerable to predation by standing out from the crowd (Jarvis 2006), though a non-typical call could also reduce identification as a prey item by the predator, counterbalancing this effect. Another potential selective pressure against vocal learning is that while flexible vocal expression can allow more advanced communication, it can also result in accidental social separation. For example, rapid divergence of vocalization, such as an isolated African elephant learning to imitate truck sounds (Poole et al. 2005), could negatively affect the fitness of individuals and prevent reintegration into the group. On the other hand, this vocal divergence could introduce cultural barriers or shibboleths that increase the likelihood of allopatric speciation, potentially conferring adaptive advantages to the lineage by increasing the rate of divergent evolution. Additional sexual selection mechanisms are also likely. For example, in songbirds, fish and whales, vocal learning is primarily used during courtship and breeding (reproductive advertisement), with many songbird and fish species losing vocal plasticity seasonally (Nottebohm and Liu 2010; Forlano et al. 2015).

Taken together, the tradeoffs outlined in the previous paragraph suggest that the ability to remember, recognize, and reproduce calls may only become beneficial in large, dynamic social groups (Sewall et al. 2016). This large-group hypothesis is complementary to our acoustic advantages hypothesis, because large groups are more likely to need efficient signaling systems to coordinate movements (e.g. complex flight formations of birds or bats), providing an additional selective pressure towards entrainment and vocal learning. More generally, acoustic advantages of grouping and synchronized movement in fish could have contributed to overcoming the challenges and tradeoffs of social living, a critical step in the evolution of vocal learning. Social group bonding and unconscious structuring (synchronization) of group movement during migration or long-distance travel could have contributed to the evolution of vocal learning by stimulating responsiveness to rhythmic auditory input.

Finally, it is important to note again that we are not proposing that the neural circuitry and gene factors used to synchronize movement necessarily lead to vocal learning, but that the neural circuitry and genetic factors used in synchronization, which are ancient and widespread, makes the convergent evolution towards vocal learning more likely (Fig. 1). Neuroanatomical structures and genetic factors that may have evolved in fish due to acoustic advantages are part of the deep homology shared by all tetrapods, which could account for the strong similarities in modern avian and mammalian entrainers and vocal learners (Jarvis et al. 2000; Feenders et al. 2008).

### From Rudimentary Entrainment to High-Level Vocal Learning

Even if there is a deep homology going back to fish or beyond for the gene structures, social interactions, and neural circuits predisposing the evolution of entrainment and vocal learning (Scharff and Petri 2011), the modern expression of vocal learning in mammals and birds depends on direct, monosynaptic projection from telencephalic vocal nuclei (Merker et al. 2015) that exceeds analogous capacities in fish (Forlano et al. 2014; Goodson and Bass 2002; Kittelberger and Bass 2013). There is theoretical evidence suggesting that these telencephalic connections that functionally define the current understanding of vocal learning did not arise de novo (Arbib 2011), and general evidence that some of the framework for sensory-guided motor learning has deep roots (Forlano et al. 2014; Goodson and Bass 2002; Kittelberger and Bass 2013; Scharff and Petri 2011). In the case of hominids (Arbib 2011) and other vocal learners (Feenders et al. 2008), gestural control circuits may have collateralized vocal control regions. Since other tetrapods have a sensory-motor arrangement similar to the human “homunculus”—in other words that the brain area representing the forelimb is directly adjacent to the area that controls facial and vocal articulation (larynx/syrinx)—highly developed auditory-motor circuits that evolved partially for their acoustic advantages could have been easily co-opted for vocal control. Songbirds, parrots, and hummingbirds have cerebral vocal learning nuclei adjacent to discrete brain areas that are active during limb and body movements (Feenders et al. 2008).

According to the acoustic advantages hypothesis, synchronized locomotion based on ISLR processing includes mutual adjustments based on auditory cues (e.g. finbeats, wing-flapping, footsteps). Mutual adjustments of movements are similar, or even functionally identical, to mimicking. Because neurons in the telencephalon are plastic and may change function (Haubensak et al. 2004; Temple 2001), circuits initially involved in the processing of ISLR could have switched functions to serve sound production machinery (i.e. mouth, beak, tongue, larynx, syrinx) instead of the forelimbs. This hypothesis is compatible with the fact that new structures unique to vocal learners are telencephalic, providing a possible explanation for how telencephalic areas achieved direct connections with vocalization centers in vocal learners (Jarvis 2004). This hypothesized switch of auditory-motor control circuits is more parsimonious than previous suggestions of transmission of neurons from visual tasks (gestures) to the auditory channel (Arbib 2011), because coupling would remain within auditory channels.

### Future Studies

It appears that ISLR allows human dyads and groups to unconsciously synch their locomotion (Nessler and Gilliland 2009; Nessler et al. 2011, 2013) and facilitates coordinated movement in fish (Pitcher et al. 1976; Pitcher 2001). Despite strong observational and theoretical evidence that sound plays a role in synchronization in many animal groups, there has been little experimental testing of this mechanism (Ravignani et al. 2014). Based on the evidence presented in this paper, ISLR may serve a signaling function to achieve synch in multiple animals groups including frogs, cetaceans, bats, and birds (Cook et al. 2013; Condro and White 2014; Wilson and Cook 2016; Ravignani et al. 2014). One way to test this hypothesis could be to investigate how animal groups synchronize movements when they are deprived of auditory cues, *e.g*. by using masking sound. Additionally, while synchronized movement is clearly beneficial for the group, it could incur costs to individuals, and there are likely internal physiological rewards such as dopamine release that reinforce this behavior (Larsson 2012a; Pinker 1997). In zebrafish, significant increases in the brain levels of dopamine have been reported during the period of gradually increasing schooling-related behavior (see Roberts et al. 2013 for review). Monitoring gene expression or hormonal response of animals in groups when exposed to ISLR would shed light on the generality of this phenomenon (i.e. do the same genetic factors and hormonal mechanisms encourage synchronization in phylogenetically distant lineages). There is already evidence that hearing music has measurable effects on animal behavior and brain chemistry (Panksepp and Bernatzky 2002; Meehan et al. 2018) and it would be of interest to investigate if rhythmic and un-rhythmic ISLR influence the release of dopamine in humans and other animals in similar or dissimilar ways.

Another testable hypothesis is that fish species that are particularly skilled at synching their locomotion (e.g. obligate schooling fish such as herring) may express genes that converge in some way with vocal learning species. As described earlier, there is evidence of common genetic factors (e.g. *FOXP2*) among zebra fish and vocal learners (Condro and White 2014; Lai et al. 2001; Wohlgemuth et al. 2014; Roberts et al. 2013; Scharff and Petri 2011), and a broader analysis of genetic factors in schooling and non-schooling fish within and among species could more definitively test this hypothesis. The hypothesis that fish near one another would synchronize gill-breathing could be observationally tested with visual or sound recordings. We predict that when situational awareness is the primary pressure (e.g. when fish are at rest), nearby fish would synchronize respiration, particularly if predators were nearby. Concerning the communicative function of ISLR, if the pre-movement vocalization of herring or the vocalization of migratory birds is used for synchronization rather than just a neutral consequence of respiration, we would predict that individuals would vocalize less in isolation than in a group for the same level of exertion. A comparative study of vocal learning abilities in bird species in relation to their abilities to fly in synchronized flocks would also be of interest. Evaluating the acoustic advantages from locomotor-respiratory coupling (such as possible reduction of masking) could elucidate associations between forelimb motor processing and vocal communication in vertebrates. Acoustic principles in moving animal groups are scarcely investigated and we suggest that further study of associated physics and neural processes is critical to understanding how synchronized behavior originally evolved and also to recognize how and why extant animal groups and species differ in this respect.

In conclusion, there are multiple lines of evidence that acoustic advantages influenced the evolution of respiratory-locomotor linkages and complex synchronized movement (schooling), and that these capacities contributed to the emergence of social grouping, in vertebrates, and eventually in a few lineages to vocal learning. This acoustic advantages hypothesis is not mutually exclusive to the hypothesis that synchronization of movement increases social connection in groups of animals. Rather it complements the social connection hypothesis and suggests a possible evolutionary mechanism selecting for social behavior and occasionally vocal learning.
